# Epidemiology of Bloodstream Infections at a Cancer Center

**DOI:** 10.1590/S1516-31802000000500004

**Published:** 2000-09-01

**Authors:** Eduardo Velasco, Luiz Claudio Santos Thuler, Carlos Alberto de Souza Martins, Márcio Nucci, Leda Maria Castro Dias, Vânia Maria da Silva Castro Gonçalves

**Keywords:** Bloodstream infection, Cancer, Epidemiology, Micro-biology, Infecção da corrente sangüínea, Câncer, Epidemiologia, Microbiologia

## Abstract

**CONTEXT:**

Cancer patients are at unusually high risk for developing bloodstream infections (BSI), which are a major cause of in-hospital morbidity and mortality.

**OBJECTIVE:**

To describe the epidemiological characteristics and the etiology of BSI in cancer patients.

**DESIGN:**

Descriptive study.

**SETTING:**

Terciary Oncology Care Center.

**PARTICIPANTS:**

During a 24-month period all hospitalized patients with clinically significant BSI were evaluated in relation to several clinical and demographic factors.

**RESULTS:**

The study enrolled 435 episodes of BSI (349 patients). The majority of the episodes occurred among non-neutropenic patients (58.6%) and in those younger than 40 years (58.2%). There was a higher occurrence of unimicrobial infections (74.9%), nosocomial episodes (68.3%) and of those of undetermined origin (52.8%). Central venous catheters (CVC) were present in 63.2% of the episodes. Overall, the commonest isolates from blood in patients with hematology diseases and solid tumors were staphylococci (32% and 34.7%, respectively). There were 70 episodes of fungemia with a predominance of Candida albicans organisms (50.6%). Fungi were identified in 52.5% of persistent BSI and in 91.4% of patients with CVC. Gram-negative bacilli prompted the CVC removal in 45.5% of the episodes. Oxacillin resistance was detected in 26.3% of Staphylococcus aureus isolates and in 61.8% of coagulase-negative Staphylococcus. Vancomycin-resistant enterococci were not observed. Initial empirical antimicrobial therapy was considered appropriate in 60.5% of the cases.

**CONCLUSION:**

The identification of the microbiology profile of BSI and the recognition of possible risk factors in high-risk cancer patients may help in planning and conducting more effective infection control and preventive measures, and may also allow further analytical studies for reducing severe infectious complications in such groups of patients.

## RESUMO

**CONTEXTO:**

Os pacientes com câncer estão expostos a um maior risco de infecções da corrente sangüínea, as quais são a principal causa de morbidade e mortalidade intra-hospitalar.

**OBJETIVO:**

Descrever as características epidemiológicas e a etiologia das infecções da corrente sangüínea em um hospital de câncer.

**TIPO DE ESTUDO:**

Estudo descritivo.

**LOCAL:**

Centro de Oncologia Terciária.

**PARTICIPANTES:**

Durante um período de 24 meses todos os pacientes hospitalizados e com infecções da corrente sangüínea, clinicamente significante, foram avaliados de acordo com diversos fatores clínicos e demográficos.

**RESULTADOS:**

O estudo compreendeu 435 episódios de infecções da corrente sangüínea (349 pacientes). A maioria dos episódios de infecções da corrente sangüínea ocorreu em pacientes não-neutropênicos (58,6%) e com menos de 40 anos de idade (58,2%). Houve uma maior ocorrência de infecções unimicrobianas (74,9%), de episódios nosocomiais (68,3%) e de origem indeterminada (52,8%). Cateteres venosos centrais estiveram presentes em 63,2% dos episódios. Os estafilococos foram os principais patógenos isolados em pacientes com doenças hematológicas e tumores sólidos (32% e 34,7%, respectivamente). A Candida albicans foi o microorganismo predominante nos 70 episódios de fungemia. Os fungos foram identificados em 52,5% das infecções da corrente sangüínea persistentes e em 91,4% das infecções em pacientes portadores de CVC. Bacilos Gram-negativos foram responsáveis pela retirada dos Cateteres venosos centrais em 45,5% dos episódios. Uma resistência à oxacilina foi detectada em 26,3% dos Staphylococcus aureus e em 61,8% dos Staphylococcus coagulase-negativa. Não foi evidenciado nenhuma cepa de enterococos resistente à vancomicina. A terapia antimicrobiana inicial foi considerada apropriada em 60,5% dos casos.

**CONCLUSÃO:**

A identificação do perfil microbiológico das infecções da corrente sangüínea e o reconhecimento dos possíveis fatores de risco em pacientes oncológicos de alto-risco poderiam ajudar no planejamento e na condução de medidas mais efetivas na prevenção e controle das infecções, além de permitir a elaboração de estudos analíticos que busquem reduzir as graves complicações infecciosas neste grupo de pacientes.

## INTRODUCTION

The improvement in long-term survival of immunocompromised patients with neoplastic diseases and those undergoing organ and bone marrow transplants has produced a population of patients even more susceptible to infections.^1^ For this reason, the infectious complications consequent to the immunosuppressive therapy have become the major causes of morbidity and mortality in this high-risk group of patients.^2^

The underlying disease, as well as the neutropenia and other risk factors, have altered substantially the epidemiology of infections, allowing the development of opportunistic infectious processes at some point during the immunosuppressive therapy.^3^ The objective of this study was to describe the epidemio-logical characteristics and the etiology of bloodstream infections (BSI) at a referral cancer center.

## METHODS

The Hospital do Câncer is a 206-bed tertiary oncology care center including medical/surgical intensive care and bone marrow transplantation (BMT) units. It is the major hospital of the National Cancer Institute complex in Rio de Janeiro, Brazil.

All hospitalized patients with an underlying cancer and those submitted to BMT with clinically significant BSI were eligible for the study. Patients were selected from the daily microbiology reports and all medical records were reviewed by two infectious disease specialists. All blood cultures from BMT patients were drawn through a central venous line (CVC), but other services did not specify which ones were obtained through a peripheral vein or a CVC. Nevertheless, there was a tendency among house officers to always ask nurses to obtain two sets of blood cultures specimens, with one of them drawn from the CVC line if it was available. Clinical and epidemiological factors were evaluated when the first positive blood specimen was obtained and included patient demographics, underlying disease, primary tumor site, clinical stage of cancer, performance status according to the Karnofsky score,^4^ duration and severity of neutropenia, use of H_2_ receptor antagonists or antacids, presence of CVC, total parenteral nutrition (TPN), corticosteroid therapy,^5^ hyperglycemia (serum glucose level >200 mg/dl or therapy with an oral hypoglycemic agent or insulin). Exposure to radiotherapy or chemotherapy, weight loss of more than 10% and prior surgical procedure were considered when they occurred over the preceding 30 days. The exposure to antibiotics was referred to when it had been used for more than three days during the preceding two weeks. The appropriateness of the empirical regimen therapy (before the index blood culture result was known) was correlated to the in-vitro susceptibility of the blood culture isolates and prior to any change in regimen made as result of growth in that culture. The therapy was deemed appropriate if the etio-logic microorganisms were susceptible to at least one of the drugs used. The clinical or laboratory response to the initial antibiotic regimen was not analyzed.

The infectious episode was defined as a clinical illness associated with the isolation of microorganisms from one or more blood cultures from the same patient. We used the day of the first positive blood culture as the definition criterion for the starting point of the episode. The end of the episode was defined as the point in time when all antibiotics were discontinued and the patient had no clinical or bacteriological evidence of infection, or at the patient's death if it occurred during the infectious episode. Patients could reenter the protocol only after they had been “off-study”, and if an interval of at least 14 days had elapsed without any sign or symptoms of infection since the preceding episode. The case-fatality rate was defined as the number of deaths from the BSI divided by the total number of BSI episodes. Death was considered only if occurring within the infectious episode.

Fever, infection and true BSI were defined according to Sickles, et al.^6^ and the Center for Disease Control (CDC).^7^ In cases with isolation of skin contaminants (e.g. Bacillus species, coagulase-negative staphylococci, diphtheroids, micrococci, or Propionibacterium species), the physician had to consider this clinically significant and immediately start antimicrobial therapy. An episode was interpreted as hospital-acquired if it occurred after 48 hours of admission or following a medical procedure, regardless of the length of hospitalization. The source of infection was delineated according to the CDC criteria.^7^ Primary BSI was related to an infection for which there was no other documented source. All episodes of BSI related to CVC were classified within this category. Secondary BSI were those episodes that developed subsequent to a clinical or laboratory documented site of infection with the same organisms.

Neutropenia was defined as an absolute neutro-phil count ≤ 1000/mm^3^. Polymicrobial episodes refer to infection in which more than one different species of organisms were isolated in a single or in a separate blood culture specimen within the same episode. Episodes were considered transient when organisms were isolated within the first 48 hours, and persistent when the blood cultures remained positive for more than 48 hours.

Identical blood culture methods were used during the study period. Blood specimens were collected and placed in culture bottles of brain-heart infusion broth and supplemented with 0.03% sodium polyanetholsulphonate as an anticoagulant. Blind subcultures were performed after 6h to 24h of incubation at 37°C using blood agar, Sabouraud dextrose agar, and brain-heart infusion agar. Quantitative blood cultures were not performed. The identification of species and susceptibility tests were performed according to the guidelines of the National Committee for Clinical Laboratory Standard (NCCLS)^8^ at the time the study was performed. Since there have been changes in the dilution recommendations by the NCCLS, not all tests correspond to the current guidelines. All negative bottles remained incubated for 30 days. Positive blood cultures for yeast were further processed in the mycological laboratory. Yeasts were identified on the basis of morphology and biochemical characteristics (API 20C; Biomérieux AS, Marcy l'Etoile, France). Non-yeast fungi were identified on the basis of morphology.

Data analysis was performed using the EpiInfo computer program (Epi 6.03; Center for Disease Control and Prevention, USA). Differences in proportions between categories were calculated using Chi-square analysis. P values <0.05 were considered statistically significant.

## RESULTS

From January 1, 1993 through December 31, 1994 the microbiology laboratory processed 10,686 blood culture sets. Overall, 1164 sets were positive for bacteria or fungi. Of these, 1037 (89%) were clinically significant for the study (127 were excluded: 62 were classified as contaminants, 42 were unavailable or had incomplete medical records, 17 had no underlying malignant disease or the patients were not BMT recipients).

During the study period, 11861 patients were admitted to the hospital, with a rate of 87.4 true positive blood cultures per 1000 admissions. Of these, 6438 (54%) were in surgical wards. The median age of patients was 34 years (range: 0-92 years). The average length of hospital stay was 38 days. There were 435 true episodes of BSI involving 349 patients. The median duration of hospitalization was 11 days from admission to the first positive blood culture (range: 30-142). Twenty episodes (4.6%) occurred before the hospital admission (median duration: 7 days) and 415 episodes (95.4%) after the hospital admission (median duration: 12 days).

Among the BSI episodes there was a similar distribution of males and females (52.4% and 47.6%, respectively). In 297 episodes (68.3%) the infection was hospital-acquired. Two hundred and fifty-three episodes (58.2%) occurred in patients younger than 40 years, who had higher frequency of acute leukemia (34%) and use of CVC (69.4%) when compared to patients older than 40 years (20% vs. 41.2%, respectively; P < 0.05). Table 1 shows that the episodes were proportionately more common in non-surgical services (82.8%). Of these, 32.4% were in the hematology ward. Almost 42% of solid tumors were disseminated at the moment of the first positive blood culture, with a predominance of gastrointestinal (16%), head and neck (12.4%) and central nervous system (10.8%) tumors.

**Table 1:** Distribution of the 435 bloodstream episodes according to hospital services and underlying diseases

Services	Episodes	(%)
**Non-surgical**	**360**	**82.8**
Hematology	141	32.4
Oncology	80	18.4
Pediatric	63	14.5
BMT	63	14.5
Radiotherapy	13	3.0
**Surgical**	**75**	**17.2**
Abdominal	30	6.9
Head and neck	12	2.8
Genitourinary	10	2.3
Central nervous system	09	2.1
Others *		
**Underlying diseases**		
**Solid tumors**	**194**	**44.6**
Disseminated	81	41.8
Local disease	113	58.2
**Hematology diseases**	**241**	**55.4**
Non-Hodgkin lymphoma	66	27.4
Acute myeloid leukemia	60	24.9
Acute lymphocytic leukemia	32	13.3
Hodgkin disease	20	8.3
Chronic myeloid leukemia	19	7.8
Myelodysplastic syndrome	15	6.2
Multiple myeloma	12	4.9
Aplastic anemia (post-BMT)	09	3.7
Chronic lymphocytic leukemia	07	2.9
Fanconi's anemia (post-BMT)	01	0.4

BMT: Bone marrow transplantation

* Chest: n = 5 (1.1%); Gynecology: n = 5 (1.1%); Bone and skin/soft tissue: n = 2 (0.5%); Breast: n = 2 (0.5%).

Fifty-seven episodes (13.1%) occurred within the first 30 days after the BMT. The median age of these patients was 26 years (range: 9-49 years), and 27 of them (57.4%) had acute or chronic myeloid leukemia.

The evaluation of the occurrence of the BSI episodes according to the moment of the treatment or clinical stage of the underlying disease, showed that 145 episodes (33.3%) developed while the patients were in remission/induction chemotherapy. The remaining episodes occurred while the disease was in relapse (n = 57), in complete remission (n = 26), in partial remission (n = 22), or stable (n = 20).

Nearly 75% of the episodes were unimicrobial (Table 2). The origin could not be determined in 230 episodes. The BSI were considered secondary in 113 episodes (26%), with the respiratory tract as the main focus (34,5%). Most of the episodes had a transient characteristic (74.2%). Among episodes of unimicrobial BSI the associated case-fatality rate was 32.5%, and among polymicrobial infections 38.5% (P = 0.25).

**Table 2:** Clinical characteristics of 435 bloodstream infection episodes

Blood culture characteristics	Episodes	(%)
Unimicrobial	326	74.9
Polymicrobial	109	25.1
Transient	323	74.2
Persistent	112	25.8
**Sources of infection**		
Primary infection	322	74
Unknown	230	71.4
Cvc-related	92	28.6
Secondary infection	113	26
Respiratory tract	39	34.5
Non-surgical skin abscess	18	15.9
Surgical site	17	15
Genitourinary tract	15	13.3
Intra-abdominal	07	6.2
Other*	17	15
**Neutrophil evaluation**		
Cell counts		
≤ 100/mm^3^	103	23.7
101 to 1000/mm^3^	77	17.7
> 1000 cells/mm^3^	255	58.6
Cell counts trend in the first 72 h		
Rising	78	17.9
Decreasing	121	27.8
Stable	236	54.3
**Platelets evaluation**		
Cell counts (cells/mm^3^)		
> 100,000	235	54.0
20,000 to 99,000	127	29.2
< 20,000	73	16.8

CVC: Central Venous Catheter;

* Tumor necrosis (n = 5), peri-rectal or anal region (n = 5), esophageal and gastrointestinal tract (n = 4), oropharynx (n = 3).

The source of unimicrobial episodes was primary in 245 (75.2%) and secondary in 81 (24.8%). Most of the unimicrobial episodes (76%) were transient and 57.6% of them occurred in patients with CVC. The majority of the 109 polymicrobial infections occurred among non-surgical and non-neutropenic patients (77% and 63.3%, respectively), with a predominance of solid tumors (45%) and lymphoma (16.5%) as the underlying diseases. In 70.6% of those episodes the origin was primary and in 68.8% transient. There was a higher number of CVC among patients with polymicrobial episodes than those with unimicrobial episodes (79.8% vs. 57.7%; P < 0.0001).

The median neutrophil count at the onset of infection was 2200/mm^3^ (range, 0 to 232,000) for the entire group, while for the neutropenic group it was 100/mm^3^ (range, 0 to 1000). The median duration of severe neutropenia (≤100/mm^3^) was 3 days, ranging from 1 to 46 days. The highest distribution of episodes (58.6%) occurred among patients whose neutrophil counts were above 1000/mm^3^. In 54.3% of the episodes the cell counts remained stable in the first 72 hours. The platelet counts were below 20000/mm^3^ in 16.8% of the episodes.

The majority of the episodes occurred among patients with poor performance status (88.7%), use of chemotherapy or radiotherapy (72%) and on antibiotics (64.1%) (Table 3). Pulmonary infiltrates were seen on chest radiography in 118 episodes (54 unilateral and 64 bilateral). Radiological study reports were not available for 158 episodes, but physicians’ notes did not mention any clinical finding suggestive of pulmonary infection. For the purpose of the study, these episodes were considered not to have lower respiratory tract infection.

**Table 3:** Characteristics of 435 bloodstream episodes. Distribution of potential risk factors according to the first positive blood culture

Risk factors	Episodes	(%)
Previous surgery	64	14.7
Weight loss >10%	219	50.3
Remote infection	174	40.0
Use of antibiotics	279	64.1
Use of anti-fungal agents	131	30.1
Chemotherapy	279	64.1
Radiotherapy	35	8.0
Corticosteroid	137	31.5
Total parenteral nutrition	55	12.6
Antacids/ H_2_ blockers	181	41.6
Hyperglycemia	42	9.7
Hypotension	101	23.2
Pulmonary infiltrates	118	27.1
Performance status: score 10-40	386	88.7
Central venous catheter	275	63.2

Three hundred and thirty-three patients (76.6%) were febrile and, in most of them (65.7%), the fever lasted longer than 48 hours. However, 102 patients (23.5%) were afebrile or hypothermic at the time of the first positive blood culture.

Indwelling intravascular catheters were present in 275 episodes (63.2%). Nearly 82% of these episodes occurred in non-surgical services and 59.3% of these patients had hematological diseases. One hundred and seventy-three CVC (63%) were introduced for long-term use purpose, of which 145 (84%) were of Hickman or Broviac type. Non-cuffed short-term catheters were present in 102 episodes.

Among the patients with CVC, 135 (49%) had their catheters removed during the infectious episode. Despite this, in 13 (10.6%) the blood cultures remained positive. Twelve episodes could not be evaluated because no cultures were obtained after the CVC removal. Persistent fever or positive cultures in the presence of adequate antimicrobial therapy accounted for 54% of all reasons for CVC removal. However, a tunnel infection motivated the removal in 16.3% of episodes. Among those episodes in which the catheter was removed, there was a predominance of Gram-negative bacilli (45.5%), followed by Gram-positive bacteria (25.9%) and fungi (20.9%). On the other hand, for those episodes with CVC retention, the Gram-positive prevailed (41.4%), succeeded by Gram-negative bacteria (34.3%) and fungi (8%). This difference was statistically significant (P < 0.05). Fungi accounted for 52.5% of the persistent infections, followed by Gram-negative bacilli (30%). The majority of the patients with persistent blood cultures (89%) had an intravenous central line placed.

Table 4 lists the 584 organisms cultured during the study. There was a similar proportion of Gram-negative and Gram-positive organisms (45% and 42.1%, respectively). Coagulase-negative staphylococci and *Staphylococcus aureus* were isolated in 17.6% and 10.6% of cultures, respectively. These organisms were associated with a CVC in 56.9% of the episodes. Overall, fungal pathogens represented 12.8% of cultures with a predominance of Candida albicans (50.6%). Main organisms responsible for BSI in patients with hematology diseases were coagulase-negative *Staphylococcus* (19.5%), *Staphylococcus aureus* (12.4%), *Pseudomonas spp*. (12%), streptococci (7.4%), *Candida albicans* (5.9%), *Candida* non*-albicans* (3.4%) and other fungi (2.8%). Among patients with solid tumors, coagulase-negative *Staphylococcus* (23%), *Staphylococcus aureus* (11.7%), *Klebsiella spp.* (8.3%), *Enterobacter spp*. (7.9%), *Pseudomonas spp*. (7.8%), *Candida albicans* (6.8%) and *Candida non-albicans* (5.6%) were the pathogens most frequently recovered from blood.

**Table 4:** Main pathogens responsible for 435 episodes of bloodstream infections at the Hospital do Câncer, 1993-1994. Total number of organisms: 584

Microorganisms	No. of isolates (%)
Fermentative Gram-negative bacilli	142 (24.3)
*Klebsiella spp*	42
*Enterobacter* spp.	40
*Serratia* spp.	24
*Escherichia* coli	22
*Proteus* spp.	03
*Citrobacter* spp.	04
Others	07
Non-fermentative Gram-negative bacilli	121 (20.5)
*Pseudomonas aeruginosa*	42
*Pseudomonas spp.*	23
*Acinetobacter spp.*	29
*Stenotrophomonas maltophilia*	11
*Flavobacterium spp.*	08
*Alcaligenes spp.*	02
*Shewanella putrefaciens*	02
Others	04
Coagulase-negative staphylococci	122 (20.7)
*Staphylococcus epidermidis*	103
Others	19
Staphylococcus aureus	62 (10.6)
Group D enterococci	21 (3.6)
Streptococci	41 (7.0)
“Viridans” group	16
*Streptococcus pneumoniae*	10
*Streptococcus pyogenes*	04
*Streptococcus agalactiae*	01
Others	10
Fungi	75 (12.8)
*Candida albicans*	38
*Candida* spp.*	26
*Hansenula* spp.	01
*Rhodotorula* spp.	02
*Wangiella* spp.	02
*Fusarium* spp.	06

* *Candida tropicalis* [n = 4], *Candida guilhermondi* [n = 4], *Candida parapsilosis* [n = 4], *Candida glabrata* [n = 3], *Candida stellatoidea* [n = 1], *Candida* spp. [*n* = 10].

Gram-negative rods were the only group of organisms isolated in 150 episodes (34.5%), but concomitantly with fungi in 43 episodes (9.8%). Gram-positive cocci were the only pathogens recovered in 183 episodes (42.1%), but were associated with Gram-negative organisms in 44 episodes (10.1%). The case-fatality rate for Gram-negative BSI was 36.2% and for Gram-positive infections 25%.

There were 70 BSI fungal episodes, and the majority of them (91.4%) had a CVC associated with the BSI. Only 14 patients (20%) had fungi isolated from another site. The occurrence of fungemia in patients hospitalized in surgical services was higher than in non-surgical services (21.3% and 15%, respectively). The mortality rate among these patients with fungemia was 41.4%.

The antibiotics most commonly used alone or in combination for empirical therapy of BSI in neutropenic patients were ceftazidime (98%), amikacin (89%), vancomycin (62.2%), imipen-cilastatin (51%), ciprofloxacin (40.2%), and amphotericin B (33.4%), while in non-neutropenic patients were ceftriaxone (82,2%), amikacin (81%), ciprofloxacin (48.7%) and vancomycin (39.6%). The overall susceptibilities of fermentative and non-fermentative Gram-negative strains to some antibiotics were as follow: imipencilastatin (95.2% and 72.9%, respectively), amikacin (79.6% and 67.8%), ciprofloxacin (92.8% and 66.4%) and ceftazidime (72.8% and 69.8%). The frequency of *Staphylococcus aureus* and coagulase-negative *Staphylococcus* strains susceptible to oxacillin was 73.7% and 38.2%, respectively. There were no vancomycin-resistant enterococci.

In 274 episodes (60.5%) the initial empirical antimicrobial therapy was considered appropriate. Twenty-three episodes could not be evaluated due to incomplete medical charts. The initial therapy was further modified in 203 episodes (46.7%). Persistent fever, despite negative blood cultures, prompted modification in 163 episodes (80.3%), in 86 of which (52.7%) the infectious processes subsided. However, in the remaining 77 episodes (47.3%), the blood cultures ultimately became positive. Forty episodes (19.7%) had the initial therapy modified due to persistent positive blood cultures or presence of resistant organisms, but four of these remained with positive cultures. Patients with unimicrobial or polymicrobial BSI episodes were initially treated adequately in 60.3% and 61.4% of the cases, respectively.

## DISCUSSION

The observed 89% of true-positive cultures in our study is higher than in other reports,^9,10^ perhaps due to the different definition criteria for BSI episodes that we adopted in febrile cancer patients. A possible classification bias may have occurred in this retrospective analysis, but as shown by Carlisle, et al.^11^ physicians have difficulties in ruling out false-positive blood cultures in this high-risk group of patients. Unlike in other studies,^12–14^ we considered all positive blood cultures occurring during the same clinical infectious illness as being representative of the same BSI episode, even if multiple sources were evident, which renders difficult the comparison of our rates with these studies.

The higher proportion of nosocomial BSI was probably related to the greater risk of hospitalized cancer patients in comparison with those in ambulatory care. Nevertheless, it should be stressed that the differentiation between hospital- and community-acquired infections in oncology patients, especially among those who are severely immunocompromised, is somewhat irrelevant because most etiological organisms have an endogenous origin. As these patients are frequently hospitalized or exposed to ambulatory instrumentation and broad-spectrum antibiotics, their endogenous microbial flora change rapidly to more invasive and resistant pathogens.

Similar to other infections, the incidence of BSI increases with age and is influenced by a variety of physiological factors.^15^ However, in our study, 58.2% of the episodes occurred among patients under 40 years of age, which may be related to the large number of acute leukemia and use of CVC in this age-group.

The influence of neoplastic diseases and poor performance status as factors predisposing towards BSI has been described in many reports.^3,10,12^ In the present study, we observed that 88.7% of the episodes occurred among patients with poor performance status (score under 40), and that 50.3% of the patients had a history of significant weight loss. The observed higher proportion of BSI (82.8%) among non-surgical patients is in agreement with other studies.^12,13^ The majority of our patients were admitted to the hematology service and BMT unit and were exposed to long-term CVC, intensive chemotherapy, prolonged and severe neutropenia and broad spectrum antibiotics.

In contrast to other studies,^12,16^ we found a greater number of BSI among non-neutropenic patients. This distribution may be related to the high number of BSI among hospitalized patients in poor clinical condition and with advanced solid tumors with normal or increased neutrophil counts.

Recent studies have shown the significance of primary and transient BSI^16^ and also the role of CVC in persistent infections.^3,17^ Overall, our data showed a predominance of primary infections (74%) with a high frequency of episodes of unknown origin (71.4%). This significant finding can be attributed to the prompt institution of broad-spectrum antibiotics for febrile cancer patients, which may also explain the observed predominance of transient (74.2%) episodes in our study.

Intravascular devices are considered the main source of primary BSI.^18^ Our data showed that only 28.6% of BSI were considered CVC-related, although 63.2% of our patients had CVC. It is worth emphasizing the significant distribution of polymicrobial BSI among non-surgical (77%) and non-neutropenic patients (63.3%) and also among those with indwelling central lines (79.8%). These multiple-organism episodes are perhaps markers for the severity of the patient's underlying diseases. The high proportion of polymicrobial transient episodes (62%) among non-surgical patients may be related to the prompt removal of CVC in the presence of hypotension or the recovery of more than one organism in the first blood culture set.

Although we were not able to precisely determine the amount of blood cultures drawn through the CVC in our study, a considerable number of them were certainly obtained through these lines, mainly because of convenience at that time, and also due to our patients’ characteristics. Results from reliability studies of blood cultures drawn from indwelling catheters are conflicting, since increased rates of false-positive cultures prolong hospitalization and increase costs.^20^ Nevertheless, some authors have demonstrated the clinical utility of this procedure in hospitalized patients, because most of the time the physicians were able to determine the significance of the microorganism.^9,21,22^

In spite of the fact that the overall proportion of Gram-negative and Gram-positive organisms was similar, differences were observed for some species. In our study, as in others,^9,18,19^ staphylococci were the leading etiologic agents of BSI, notably the coagulase-negative types (122 episodes). Some investigators have pointed out the importance of Candida species and coagulase-negative Staphylococcus adherence to the catheter surface, and slime production, as risk factors for BSI.^19^ Our results showed that staphylococcal and fungal infections were associated with a CVC in 56.9% and 91.4% of the episodes, respectively, and that 89% of those patients with persistent blood cultures had intravascular central lines. Furthermore, fungi were the most frequent isolate during these persistent infections (52.5%). All these findings suggest a possible correlation between these organisms and the CVC in our population group.

Several authors^17–19^ reported a high distribution of Pseudomonas species and Stenotrophomonas maltophilia organisms in blood cultures of immunocompromised patients with CVC and showed their association with the catheter removal. Pseudomonas species and Stenotrophomonas maltophilia were isolated in 13.3% of our series and all of them were associated with the catheter removal. Overall, Gram-negative bacilli (45%) were the pathogens most frequently identified during the infectious episodes that ended up with CVC removal.

Polymicrobial sepsis is a significant problem, especially among patients with cancer.^9,22,23^ In the current study, the observed mortality rate among patients with polymicrobial BSI (38.5%) is comparable to recent reports.^9,23^ In contrast to one other study,^9^ we found a higher proportion of death in unimicrobial infections (32.5% and 15.7%), probably as a result of the clinical characteristics of our patients.

The 60.5% appropriateness of the empirical antimicrobial therapy in our study could be explained, at least in part, by the constant changes in the hospital microbial sensitivity pattern. The institution's protocol for the initial empirical therapy for febrile episodes was based in the main pathogens that had been isolated in blood cultures in the year prior to the study. In addition, our data showed a hospital-acquired BSI predominance, especially among patients with poor performance status and prior use of antimicrobial agents, which presumably had predisposed them to the development of more resistant BSI.

In contrast to other authors,^9^ we observed similar proportions of appropriate initial therapy in unimicrobial and polymicrobial infectious episodes (60.3% and 61.4%, respectively). It is interesting to note that those 203 patients who initially had negative blood cultures and persistent fever, fungi or resistant organisms were ultimately recovered in 47.3% of the episodes despite the therapeutic modification. We also identified 103 patients with persistent positive blood cultures despite antimicrobial therapy, most of them with Gram-negative and fungal organisms. The reasons for this occurrence may be multiple, but the presence of CVC, poor performance status, severe mucositis and neutropenia, which are considered strong risk factors for BSI, may have played a role in this phenomenon.^3,11^

In conclusion, the present study describes the epidemiological characteristics and the etiological microorganisms of BSI in a high-risk group of patients. Several aspects were noteworthy and consistent with the literature: the predominance of primary BSI, most of them from unknown sources; the importance of the respiratory tract as the main source for secondary BSI; the previous use of chemotherapy, antibiotics, central venous lines and poor performance status scoring as potential risk factors; the predominance of staphylococci and fungi as causative pathogens, especially among patients with CVC; the low appropriateness of initial empirical antimicrobial therapy; and the higher case-fatality rate during fungal episodes. The observed high frequency of coagulase-negative staphylococci is an unresolved problem, since physicians have difficulties in interpreting the significance of these isolates. However, in our study these episodes of BSI were associated with CVC and clinical signs of infection. The emerging trends in antibiotic resistance and their implications for empirical therapy indicate that institutions that support high-risk cancer patients should have active ongoing microbiological surveillance studies with the objective of monitoring infections due to antibiotic-resistant pathogens, in order to improve their current antimicrobial regimens. So, an understanding of the epidemiology of BSI is crucial for the implementation of strategies that may contribute to preventing and controlling these infections. Further analytical studies are needed to investigate independent risk factors for BSI.
